# HA198 mutations in H9N2 avian influenza: molecular dynamics insights into receptor binding

**DOI:** 10.3389/fvets.2024.1526600

**Published:** 2025-01-08

**Authors:** Rui Zhu, Jie Wu, Ruiying Chen, Mo Zhou, Shinuo Cao, Zhi Wu, Ligang Wang, Lei Zhang, Shanyuan Zhu

**Affiliations:** ^1^Jiangsu Agri-animal Husbandry Vocational College, Taizhou, Jiangsu, China; ^2^Jiangsu Key Laboratory for High-Tech Research and Development of Veterinary Biopharmaceuticals, Taizhou, Jiangsu, China; ^3^Jiangsu Co-innovation Center for Engineering Technology Research Center for Modern Animal Science and Novel Veterinary Pharmaceutic Development, Taizhou, Jiangsu, China

**Keywords:** H9N2 avian influenza virus, mutations at residue 198, hemagglutinin, receptor binding, molecular dynamics simulations

## Abstract

**Introduction:**

The H9N2 avian influenza virus is widely disseminated in poultry and poses a zoonotic threat, despite vaccination efforts. Mutations at residue 198 of hemagglutinin (HA) are critical for antigenic variation and receptor-binding specificity, but the underlying molecular mechanisms remain unclear. This study explores the molecular mechanisms by which mutations at the HA 198 site affect the antigenicity, receptor specificity, and binding affinity of the H9N2 virus.

**Methods:**

Using the sequence of the A/Chicken/Jiangsu/WJ57/2012 strain, we constructed recombinant H9N2 viruses, including rWJ57, rWJ57/HA_198A_, and rWJ57/HA_198T_, using reverse genetics. These variants were analyzed through hemagglutination inhibition (HI) assays, receptor-destroying enzyme (RDE) assays, enzyme-linked immunosorbent assays (ELISA) and solid-phase receptor binding assays. Additionally, molecular dynamics (MD) simulations were performed to further dissect the atomic-level interactions between HA and sialic acids (SA).

**Results:**

The results demonstrated that HA mutations significantly altered the receptor-binding properties of the virus. Specifically, rWJ57 (HA_198V_) exhibited 4-fold and 16-fold higher overall receptor-binding avidity compared to rWJ57/HA_198A_ and rWJ57/HA_198T_, respectively. Furthermore, HA_198V/T_ mutations significantly enhanced viral binding to human-type α2,6 SA receptors (*p* < 0.001), whereas the HA_198A_ mutation exhibited a marked preference for avian-type α2,3 SA receptors (*p* < 0.001). Additionally, these mutations altered interactions with non-specific antibodies but not specific antibodies, with high-avidity receptor binding mutations exhibiting reduced non-specific antibody binding, suggesting a potential novel mechanism for immune evasion. MD simulations revealed HA_198V/T_ formed stable complexes with the α2,6 SA, mediated by specific residues and water bridges, whereas HA_198A_ formed stable complexes with the α2,3 SA. Interestingly, residue 198 interacted with the α2,6 SA via water bridges but had with showed minimal direct interaction with α2,3 SA.

**Discussion:**

This study provides new insights into the molecular basis of receptor specificity, binding affinity, and antigenic drift in H9N2 viruses, highlighting the critical role of HA 198 mutations in regulating host adaptation. These findings are of great significance for H9N2 virus surveillance, vaccine development, and zoonotic transmission risk assessment.

## Introduction

H9N2 avian influenza virus (AIV) has been emerged as one of the most concerning low-pathogenic strains in China (1), posing significant challenges to both the poultry industry and public health. This virus not only co-infects with bacterial pathogens such as *Escherichia coli*, *Staphylococcus aureus*, and *Haemophilus paragallinarum* (2–4), but also serves as a genetic donor for human-infecting influenza subtypes, including H3N8, H7N9, H10N3, and H10N8 (5–9). Despite China’s ongoing H9N2 vaccination efforts for more than two decades, the virus continues to circulate widely, driven by rapid antigenic variation (1). The hemagglutinin (HA) protein, particularly its receptor-binding domain (RBD), undergoes frequent mutations that affect both antigenicity and receptor specificity (10). Among the various mutation sites in the HA protein, position 198 (H9 whole numbering system, corresponding to position 180 in H9 mature peptide numbering and position 190 in H3 numbering) has emerged as a critical focal point for understanding H9N2 virus evolution. This position, located near the receptor-binding domain, demonstrates a compelling pattern of amino acid substitutions that suggests strong selective pressure (11).

Previous research has shown that H9N2 vaccine strains commonly acquire the A198V mutation after multiple passages in specific-pathogen-free (SPF) chickens, leading to a significant reduction in hemagglutinin inhibition (HI) titers and an increase in receptor-binding activity (12–14). This mutation is also linked to the virus’s ability to evade antibody-mediated immunity, further complicating disease control efforts (15, 16). The mutation at amino acid 198 within the HA protein, which is often found at this receptor-binding site, can also influence the binding affinity of H9N2 to sialic acid receptors (17). Despite these findings, the molecular mechanisms by which mutations at the 198 site affect receptor-binding affinity remain poorly understood, underscoring the need for deeper investigation.

With recent advances in biological detection technologies, the precision of methods used to study virus-receptor interactions has significantly improved. Traditional techniques such as erythrocyte adsorption assays have been replaced by more advanced methods like solid-phase direct binding assays, glycan microarrays, surface plasmon resonance, and X-ray crystallography (18–20). However, while these biological detection methods provide valuable insights, they are often complemented by computational approaches such as molecular docking and molecular dynamics (MD) simulations, which can simulate interactions between viral proteins and receptors under natural conditions (21, 22). Although these computational techniques are widely used in drug discovery, they are underutilized in research on virus-host interactions, particularly in influenza viruses.

This study aims to investigate the molecular basis of receptor-binding alterations caused by mutations at position 198 in the HA protein of H9N2 AIV. Using the epidemic strain A/Chicken/Jiangsu/WJ57 (H9N2, WJ57) with 198 V as the backbone (23), we employed reverse genetics to generate recombinant viruses carrying V198A and V198T mutations. The effects of these mutations on receptor-binding activity were assessed experimentally, while molecular docking and MD simulations were used to analyze the interactions between the HA protein and sialic acid receptors. Our findings reveal the molecular mechanisms by which mutations at the 198 site affect receptor-binding affinity and sialic acid specificity, contributing to a better understanding of the adaptive evolution of H9N2 AIV and its antigenic variation.

## Materials and methods

### Ethical compliance

The SPF chicken embryos were purchased from Nanjing Biology Medical Factory Qian Yuan-hao Biological Co., Ltd. All animal experiments adhered to the Guide for the Care and Use for Laboratory Animals stipulated by the Ministry of Science and Technology, and followed the animal protection and management regulations of the Jiangsu Agri-Animal Husbandry Vocational College (Approval No: jsahvc-2024-60).

### Viruses and cells culture

The sequences of the H9N2 vaccine strain A/chicken/Jiangsu/WJ57/2012 (WJ57), published in the GenBank database under accession numbers KP893703 to KP893708 and KJ000709 to KJ000710, were synthesized by SYNBIO Technologies (Suzhou, China). The recombinant WJ57 strain (rWJ57) was generated using reverse genetics and stored at the Jiangsu Key Laboratory for High-Tech Research and Development of Veterinary Biopharmaceuticals. COS-1 cells were purchased from ATCC (Manassas, VA, United States), cultured in Dulbecco’s Modified Eagle’s Medium (DMEM) (Gibco, BRL, Grand Island, United States) supplemented with 10% fetal calf serum (Gemini, Woodland, CA, United States), and incubated at 37°C with 5% CO₂.

### HA gene sequencing analysis

The frequency and prevalence of residues at amino acid position 198 in HA were analyzed by comparing 4,093 full-length H9N2 HA sequences from Chinese isolates deposited in GenBank from 2013 to 2021 (up to January 1, 2023).

### Generation of recombinant H9N2 viruses

Primers for site-directed mutagenesis and homologous recombination were designed based on the HA sequence of the WJ57 virus using Primer 5.0 software (Primer-E Ltd., Plymouth, United Kingdom). Overlap PCR was used to amplify the mutant HA genes, which were then inserted into the transcriptional/expression vector pHW2000 using the ClonExpress II One Step Cloning Kit (Vazyme Biotech, Nanjing, China). After transformation into DH5α cells, 10 colonies were randomly selected, cultured, and subjected to plasmid DNA extraction (TianGen, Beijing, China). The constructs were then sequenced (GENEWIZ, Suzhou, China) to confirm their accuracy. The primers used in the amplification process are listed in Table 1.

**Table 1:** Primers used for construction of recombinant plasmids.

Primer	Primer sequence (5′–3′)
^a^pHW-HA-F	gtcgacctccgaagttgggggggAGCAAAAGCAGGGGAATTTC
^a^pHW-HA-R	ggcattttgggccgccgggttattAGTAGAAACAAGGGTGTTTTTGCC
^b^V198A-F	CCACCGATACTGCACAGACAAATCT
^b^V198A-R	AGATTTGTCTGTGCAGTATCGGTGG
^b^V198T-F	CCACCGATACTACGCAGACAAATCT
^b^V198T-R	AGATTTGTCTGCGTAGTATCGGTGG
^c^pHWvector-F	ACCCGGCGGCCCAAAATGCC
^c^pHWvector-R	CCCCAACTTCGGAGGTCGAC

a Primers employed for pHW2000 construction using a binucleotide cloning strategy to amplify the full-length cDNA of the HA gene. Lowercase letters denote the homologous arm sequences from the pHW2000 vector.

b Primers used for site-specific mutations in the HA gene, with sequences marked by a single underline indicating the nucleotides subjected to mutation.

c Primers utilized for amplifying the linearized pHW2000 vector.

As described by Zhu et al. (14), the H9N2 recombinant viruses, rWJ/HA_198A_ and rWJ/HA_198T_, were identified and rescued using the rWJ57 strain backbone. Briefly, a total of 2.4 μg of eight plasmids, mixed in equal amounts, was added to 100 μL of Opti-MEM medium (Gibco, BRL, Grand Island, United States). Then, 5 μL of X-treme GENE HP DNA Transfection Reagent (Roche, Basel, Switzerland) was introduced to the mixture, which was incubated at room temperature for 15 min. The mixture was then added to COS-1 cells cultured in 12-well plates at 70–80% confluency. The cells were incubated at 37°C with 5% CO₂ for 24 h, after which 2 μg/mL of TPCK-trypsin (Sigma, St. Louis, MO, United States) was added to each well. Following a 30-h transfection period, the supernatants were collected and used to propagate the virus in 10-day-old specific pathogen-free (SPF) embryonated chicken eggs. Finally, the rescued virus was subjected to an HA assay, and the HA genes were sequenced at GENEWIZ (Suzhou, China) to confirm the accuracy of the intended mutations.

### Antisera preparation

Antisera for the rWJ57 strain were prepared as previously described (11, 14). Briefly, 3-week-old SPF chickens were immunized twice by subcutaneous injection of 0.3 mL of an oil-emulsified, inactivated whole-virus vaccine of the rWJ57 strain, which was inactivated by adding 0.2% formalin (*v*/v) for 24 h at 37°C. Antisera were collected from the chickens 2 weeks after the booster vaccination. The negative sera used were collected from non-immunized SPF chickens as a pre-immunized control.

### Hemagglutination inhibition assay

Antisera for the rWJ57 strain were treated with cholera filtrate (Sigma-Aldrich, St. Louis, MO, United States) to remove nonspecific hemagglutination inhibitors before conducting the HI assay on three recombinant viruses: rWJ57, rWJ/HA_198A_ and rWJ/HA_198T_. The assay was performed using 4 hemagglutination units (HAU) of H9N2 and 1% (*v*/v) chicken erythrocytes (14, 24).

### Enzyme-linked immunosorbent assay

The ELISA assay was conducted according to previously described methods (14, 24). Sucrose gradient-purified viruses—rWJ57, rWJ/HA_198A_ and rWJ/HA_198T_—were diluted in PBS and seeded into Nunc-Immuno MaxiSorp 96-well plates (Corning, NY, United States) at a concentration of 16 HAU per well. Following overnight incubation at 4°C, the wells were blocked with PBS-nonfat dry milk (Beyotime, Shanghai, China). Serial two-fold dilutions of antisera against the rWJ57 virus in chickens were prepared using PBS containing 0.05% Tween 20 and then added to the wells, followed by a 2-h incubation at 37°C. After washing, a goat anti-chicken horseradish peroxidase antibody (Bioss, Beijing, China) was added, and the plates were incubated for 1.5 h at 37°C. Finally, TMB (3,3′,5,5′-tetramethylbenzidine) substrate (Beyotime, Shanghai, China) was added, and the reaction was stopped using TMB stop substrate (Beyotime, Shanghai, China).

Absorbance was measured at 450 nm using an automated ELISA plate reader (model EL311SX; Biotek, Winooski, VT, United States). The area under the curve (AUC) for each virus was calculated using GraphPad Prism 9.5 software (San Diego, CA, USA), based on virus-antibody binding above the corresponding negative control.

### Receptor-destroying enzyme assay and solid-phase binding assay

The RDE assay was performed as previously described (14, 25). Chicken erythrocytes were treated with α2-3,6,8 neuraminidase (New England Biolabs, Beverly, MA, United States) through 11 two-fold serial dilutions starting from a maximum concentration of 1,000 U/mL for 1 h at 37°C. After washing with PBS, 1% (*v*/v) solutions of the treated erythrocytes were added to 4 HAU of each virus (determined using untreated chicken erythrocytes). Agglutination was measured after 1 h of incubation.

To further evaluate the receptor-binding properties of the H9N2 viruses, we performed solid-phase binding assays using the synthetic sialyl glycopolymers Neu5Ac 2–3 Galβ1-4 GlcNAc (3′SLN)-PAA-biotin and Neu5Ac 2–6 Galβ1-4 GlcNAc (6′SLN)-PAA-biotin (GlycoTech), based on the method described by Zhang et al. (26, 27). The glycan analogs were serially diluted in PBS and added to streptavidin-coated 96-well plates (Pierce). Plates were blocked with PBS containing 2% skim milk, and 64 HA units of virus were applied per well, followed by chicken antiserum as the primary antibody. Detection was done by adding HRP-conjugated rabbit anti-chicken IgG and tetramethylbenzidine substrate, with the reaction stopped by 1 M H_2_SO_4_. Absorbance at 450 nm was measured, and all samples were tested in triplicate.

### Molecular docking and dynamics simulation

The structures of HA were predicted using the Swiss-Model and validated by Ramachandran plot analysis, with 98.2% of residues in favored regions. The protonation state of all compounds was set to pH 7.4, and 3D structures were generated using Open Babel (28). AutoDock Tools (ADT3) were used to prepare and parameterize the receptor protein and ligands. Docking grid files were constructed with AutoGrid from the sitemap, and docking simulations were performed using AutoDock Vina (1.2.0) (29, 30). The optimal pose was selected for protein-ligand interaction analysis. Finally, protein-ligand interaction figures were generated using PyMOL. The ligands used in this study were α2,6 sialic acid (SA) (6′SLN, CAS no. 501427–93-6) and α2,3 SA (3′SLN, CAS no. 501427–92-5).

MD simulations were conducted using the non-commercial version 2022.1 of Desmond/Maestro software.^1^ TIP3P water molecules were added to the systems, which were neutralized with a 0.15 M NaCl solution. After system minimization and relaxation, a 100-ns production simulation was performed using an isothermal-isobaric ensemble at 300 K and 1 bar. Trajectory coordinates were recorded every 100 ps throughout the simulation. Molecular dynamics analysis was performed using the Simulation Interaction Diagram feature of Desmond software.

### Statistical analysis

Data normality was verified using Shapiro–Wilk test. For experiments comparing both virus types and concentrations (receptor binding assays), statistical analysis was performed using two-way ANOVA with Holm-Bonferroni correction for multiple comparisons to control family-wise error rate. For single variable comparisons (HI titers and antibody binding assays), pairwise comparisons were made using Student’s *t*-test. All statistical analyses were conducted in GraphPad Prism 9.5, with adjusted *p*-values less than 0.05 considered statistically significant. All experiments were performed with three independent biological replicates (*n* = 3), with technical duplicates for each biological replicate to ensure measurement reliability. The mean values from technical duplicates were used for statistical analysis. For molecular dynamics simulations, each system was simulated for 100 ns and repeated three times with different initial velocities to ensure conformational sampling adequacy.

## Results

### Analysis of the prevalence of various residues at the position 198

**Figure 1:**
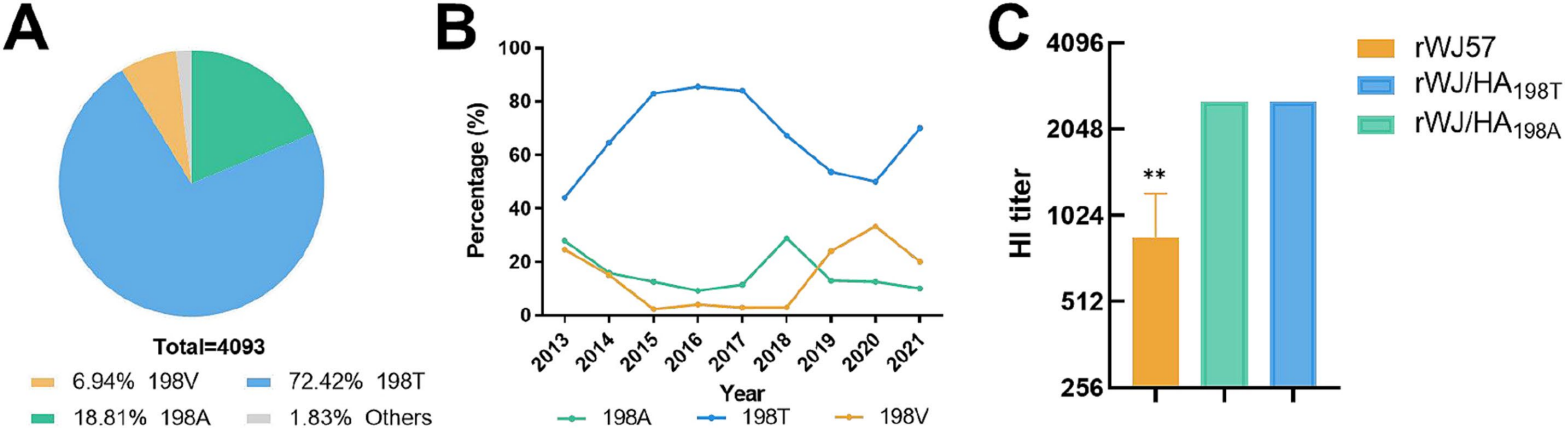
Analysis of amino acid mutations at position 198 in the HA protein of H9N2 AIV. **(A)** Distribution of HA protein variants among 4,093 analyzed sequences. **(B)** Temporal trends in the prevalence of HA protein variants 198A, 198 T, and 198 V from 2013 to 2021. **(C)** HI titers of different recombinant viruses (rWJ57 (HA_198V_), rWJ/HA_198T_, and rWJ/HA_198A_) against anti-rWJ57 sera. Data are presented as mean ± SD from three independent biological replicates (*n* = 3), with technical duplicates for each replicate. Statistical analysis was performed using Student’s *t*-test, **p < 0.001.

An analysis of 4,093 avian H9N2 AIV HA protein sequences at position 198, published in GenBank from 2013 to 2021, revealed three prevalent residues: 198 T, 198A, and 198 V. The most frequent was 198 T, representing 72.4% of the sequences, followed by 198A (20.7%), while 198 V was the least common (6.9%) (Figure 1A). Between 2013 and 2015, the frequency of the 198 T mutation steadily increased, reaching over 80% from 2015 to 2017. However, from 2017 to 2020, the prevalence of 198 T steadily declined, dropping to 70% in 2021, while the 198 V showed an inverse trend, rising in frequency and becoming dominant by 2021 (Figure 1B). The 198A frequency remained stable (10–15%) throughout the study period, with minor fluctuations.

### Rescue and identification of recombinant viruses

Following transfection of COS-1 cells with eight plasmids encoding the full H9N2 genome, the supernatants were inoculated into SPF chicken embryos. After 90 h, HA titers were measured in the allantoic fluid, and recombinant viruses with HA titers ≥2^2^ were considered positive. The HA titers for rWJ/HA_198A_ and rWJ/HA_198T_ viruses were 2^6^ and 2^8^, respectively. Sequencing confirmed that the HA genes contained the desired mutations at positions 591–594, while all other gene segments (PB2, PB1, PA, NP, NA, M, and NS) were identical to the parental rWJ57 strain.

### Mutations at HA position 198 as a key modulator of HI titers

To investigate how mutations at residue 198 influence the antigenic properties of the virus, HI assays were performed using sera from chickens infected with rWJ57. The rWJ57 virus carrying the 198 V mutation showed a three-fold lower HI titer compared to rWJ/HA_198A_ and rWJ/HA_198T_ (Figure 1C), suggesting that residue 198 plays a critical role in promoting viral escape from a polyclonal antibody binding.

### Mutations at HA position 198 affects the binding affinities to non-specific antibodies

**Figure 2:**
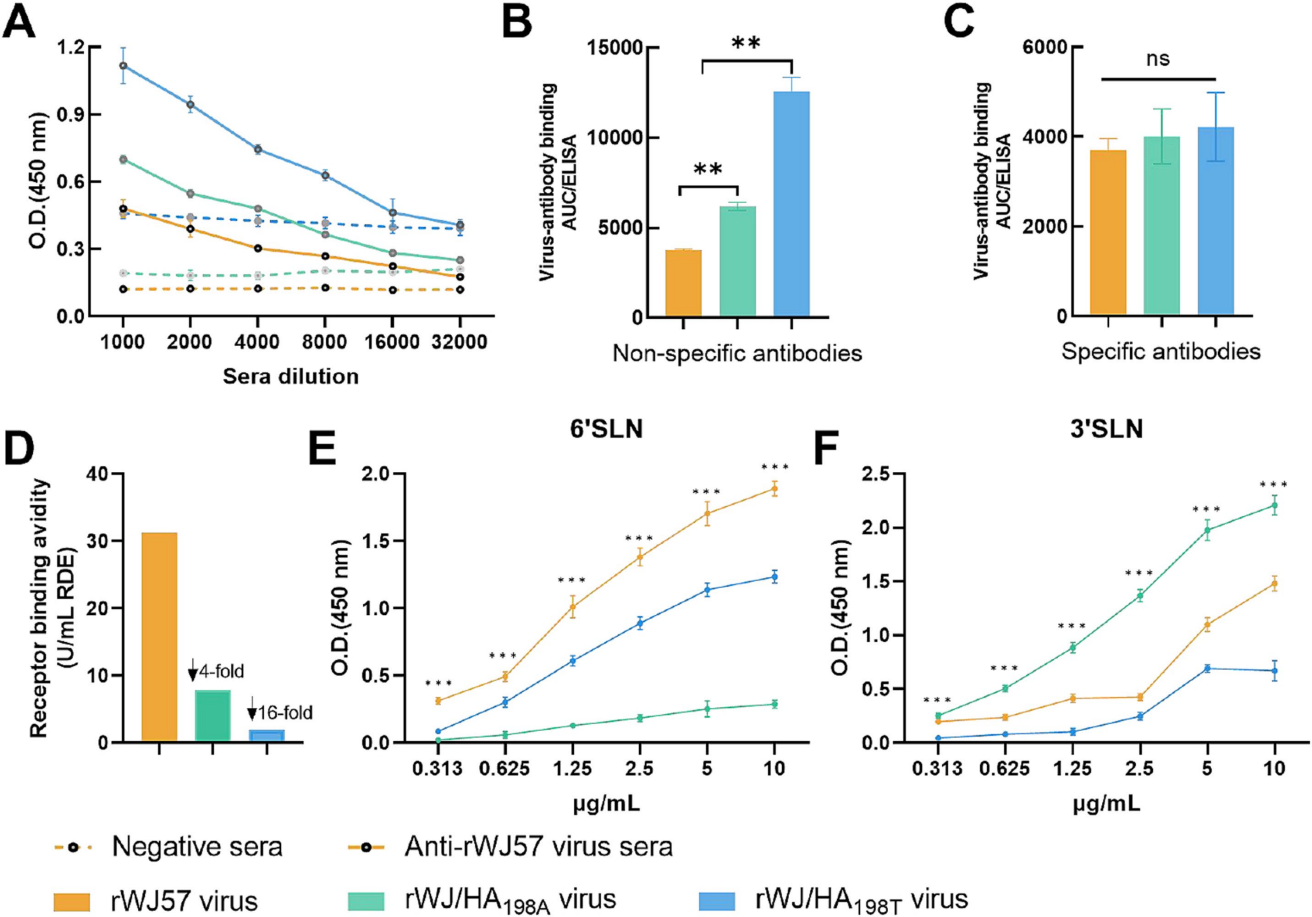
Characterization of recombinant H9N2 viruses with mutations at HA position 198. **(A)** ELISA-based antibody binding curves for negative sera (dotted lines) and anti-rWJ57 virus sera (solid lines) against three recombinant viruses. Negative control sera were collected from age-matched SPF chickens before immunization. Background signals from no-serum controls were subtracted from all measurements. **(B)** Area under the curve (AUC) of non-specific antibody binding and **(C)** specific antibody binding to recombinant viruses, measured by ELISA. The AUC values were calculated using GraphPad Prism 9.5 based on the binding curves in **(A)**, where non-specific binding represents the AUC of negative sera, and specific binding represents the difference between anti-rWJ57 sera and negative sera AUC values. **(D)** Receptor binding avidity of recombinant viruses determined by RDE assay. **(E)** Binding affinity of recombinant viruses to 6’SLN and **(F)** 3’SLN receptor analogs. rWJ57 virus (orange), rWJ/HA_198A_ virus (green), and rWJ/HA_198T_ virus (blue) were used. All experiments were performed with three independent biological replicates (*n* = 3), with technical duplicates for each biological replicate. Data points represent the mean ± SD of the biological replicates. Statistical analysis was performed using two-way ANOVA with Holm-Bonferroni correction. **p < 0.01, ***p < 0.001; NS, not significant; SLN, sialylated glycan analogs.

To explore the underlying mechanisms, ELISA assays were conducted to measure the binding affinities of specific and non-specific antibodies. The results demonstrated that the rWJ57 virus had significantly lower affinity for non-specific antibodies compared to rWJ/HA_198A_ (*p* < 0.001), while rWJ/HA_198T_ displayed significantly higher affinity than both rWJ57 and rWJ/HA_198A_ (*p* < 0.001) (Figures 2A,B). However, no significant differences were observed in the binding affinities of the three viruses to specific antibodies (Figures 2A,C).

### Mutations at HA position 198 influences the receptor binding activity and specificity

To assess how receptor binding activity affects HI titers, chicken erythrocytes were treated with α2-3,6,8 neuraminidase to modify sialic acid receptors. Stronger receptor binding was reflected by the virus’s ability to agglutinate erythrocytes treated with higher concentrations of neuraminidase. Results showed that rWJ57 exhibited receptor binding activity four-fold higher than rWJ/HA_198A_ and 16-fold higher than rWJ/HA_198T_ (Figure 2D). These findings suggest that the mutation at position 198 alters receptor binding, which primarily drives HI titer fluctuations in the H9N2 virus, rather than specific antibodies.

While the RDE assay demonstrates the virus’s binding activity for sialic acids (both α2-3 SA and α2-6 SA), it cannot differentiate the virus’s affinity for specific sialic acid types. To further explore the effect of mutations at HA position 198 on the affinity for specific sialic acids, we conducted a solid-phase binding assay. The results revealed distinct receptor preferences among H9N2 virus variants with mutations at HA position 198. For 6’SLN binding, rWJ57 demonstrated the highest binding affinity across all tested concentrations (0.313–10 μg/mL), followed by rWJ/HA_198T_, while rWJ/HA_198A_ showed the lowest affinity. The differences in binding affinity among the three viruses were statistically significant at each tested concentration (*p* < 0.001) (Figure 2E). Conversely, for 3’SLN binding, rWJ/HA_198A_ exhibited the strongest binding affinity, followed by rWJ57, while rWJ/HA_198T_ showed the lowest affinity. Similarly, significant differences in binding affinity were observed among all three viruses at each tested concentration (*p* < 0.001) (Figure 2F). These findings indicate that the amino acid at position 198 of the HA protein plays a crucial role in determining receptor binding specificity.

### Mutations at HA position 198 alter in HA protein-sialic acid receptor interactions

**Figure 3:**
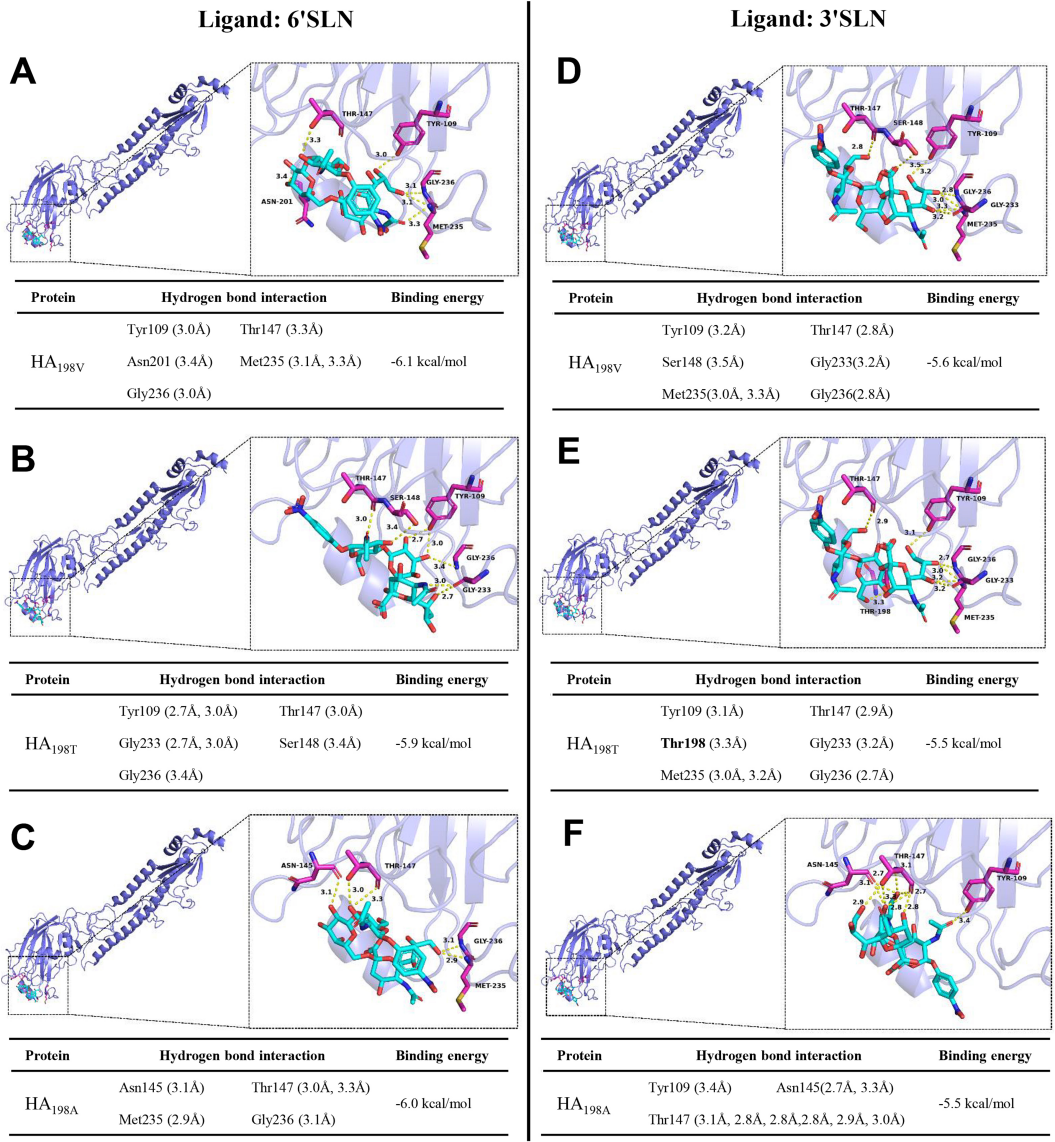
Molecular docking analysis of 6’SLN and 3’SLN with HA proteins of the recombinant viruses. **(A–C)** Docking results of 6’SLN with HA proteins: **(A)** HA_198V_, **(B)** HA_198T_, and **(C)** HA_198A_. **(D–F)** Docking results of 3’SLN with HA proteins: **(D)** HA_198V_, **(E)** HA_198T_, and **(F)** HA_198A_. Each panel displays the overall structure of the HA protein (left) with a zoomed-in view of the binding site (right). The HA protein is represented as a slate cartoon model. Ligands (6’SLN and 3’SLN) are shown as cyan sticks, with key interacting residues highlighted as magenta sticks. Nonpolar hydrogen atoms are omitted for clarity. Hydrogen bonds, ionic interactions, and hydrophobic interactions are depicted as yellow, magenta, and green dashed lines, respectively. Tables below each structure summarize the hydrogen bond interactions and binding energies. Hydrogen bond distances are indicated in Ångstroms (Å).

The α2,6 SA formed five hydrogen bond interactions with the HA protein of the rWJ57 strain (HA_198V_ protein) at amino acid positions 109, 147, 201, 235, and 236, with a binding energy of −6.1 kcal/mol (Figure 3A). It also formed five hydrogen bond interactions with the rWJ/HA_198T_ HA protein (HA_198T_ protein) at positions 109, 147, 148, 233, and 236, with a binding energy of −5.9 kcal/mol (Figure 3B), and four interactions with the rWJ/HA_198A_ HA protein (HA_198A_ protein) at positions 145, 147, 235, and 236, with a binding energy of −6.0 kcal/mol (Figure 3C).

The α2,3 SA formed six hydrogen bond interactions with the HA_198V_ protein at amino acid positions 109, 147, 148, 233, 235, and 236, with a binding energy of −5.6 kcal/mol (Figure 3D). It also formed six interactions with the HA_198T_ protein at positions 109, 147, 198, 233, 235, and 236, with a binding energy of −5.5 kcal/mol (Figure 3E), and three interactions with the HA_198A_ protein at positions 109, 145, and 147, with a binding energy of −5.5 kcal/mol (Figure 3F).

These results indicate that alterations at amino acid position 198 of the HA protein directly modify the microstructure of the receptor-binding pocket, leading to changes in the binding sites, interaction forces, torque, and binding energy with sialic acid receptors, ultimately influencing the interactions between the HA protein and the receptors.

### MD simulation

To explore the molecular mechanisms by which mutations at residue 198 influence receptor binding affinity and viral tropism, molecular dynamics (MD) simulations were employed to evaluate the structural and dynamic behavior of the HA-sialic acid receptor complexes. The simulation focused on how the HA_198V_, HA_198T_, and HA_198A_ mutations alter the interaction between the HA protein and both α2,6 SA and α2,3 SA. The key metrics analyzed were root mean square deviation (RMSD), root mean square fluctuation (RMSF), hydrogen bonding patterns, and binding stability, which provided insights into conformational changes and receptor affinity.

### MD simulation demonstrates stable binding stability between HA198V and α2,6 SA

**Figure 4:**
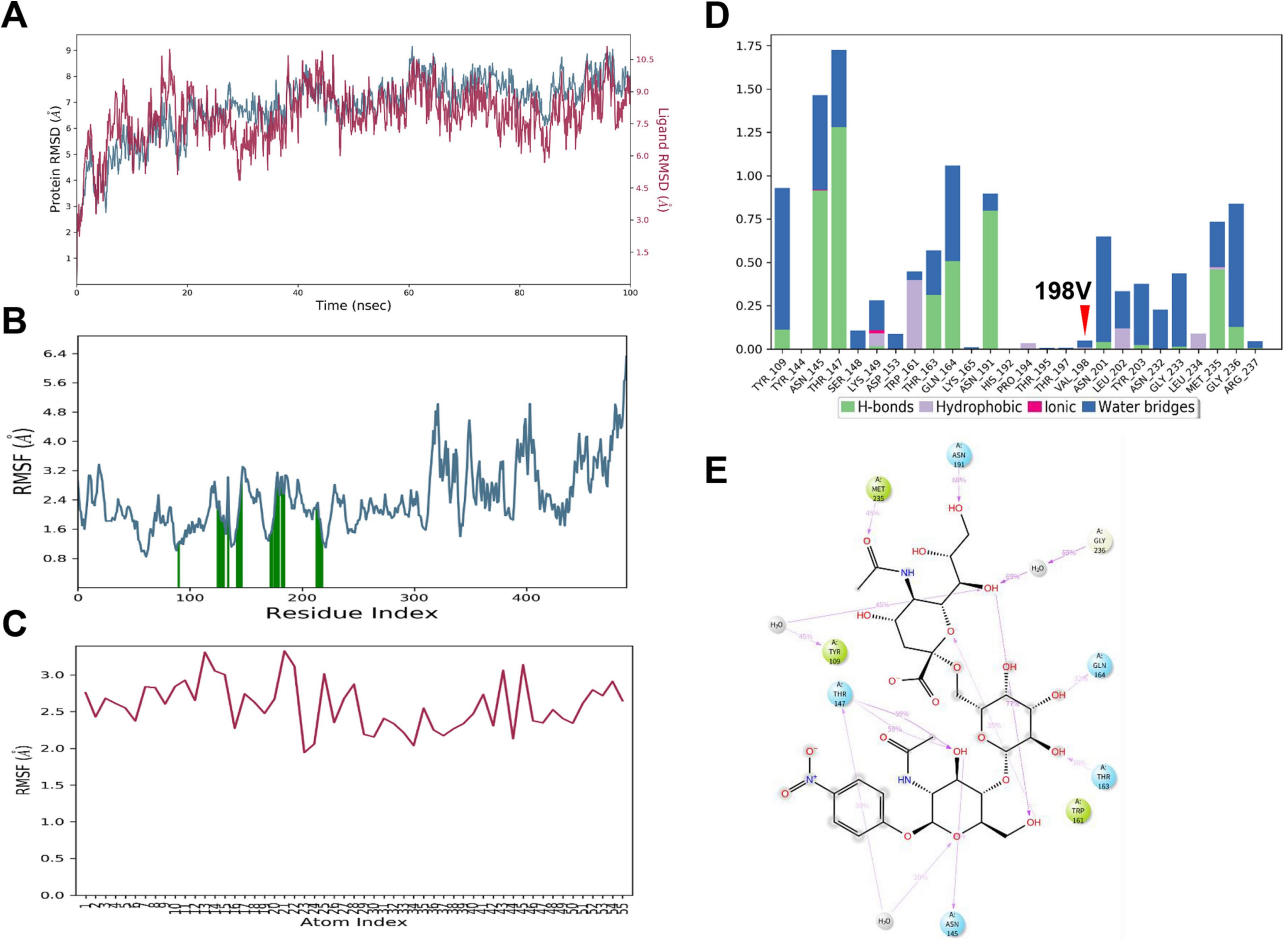
MD simulation results for 6’SLN and HA_198V_. **(A)** RMSD plot of HA_198V_ (blue) and 6’SLN (red) over 100 ns of simulation time. **(B)** RMSF plot of HA_198V_ residues, with green bars indicating regions of high fluctuation. **(C)** RMSF plot of 6’SLN atoms. **(D)** Interaction analysis between 6’SLN and HA_198V_. Bar graph shows the frequency of different types of interactions (hydrogen bonds, hydrophobic interactions, ionic interactions, and water bridges) for specific residues. The red arrow indicates the mutation site 198 V. **(E)** 2D interaction diagram of 6’SLN with surrounding amino acid residues of HA_198V_. Dashed lines represent different types of interactions.

During the simulation, the HA protein maintained a stable interaction with the α2,6 SA. The RMSD values for the HA_198V_ protein and the α2,6 SA stabilized around 7.0 Å and 9.0 Å, respectively, after initial fluctuations (Figure 4A), suggesting that the complex underwent minor adjustments before reaching a stable conformation. RMSF analysis (Figures 4B,C) revealed minimal fluctuations in key binding residues, particularly Tyr109, Thr147, Met235, and Gly236, highlighting their crucial role in maintaining the interaction’s stability.

Thr147 played a pivotal role in the binding process, forming hydrogen bonds with a high formation frequency of 118%, supported by secondary water bridge interactions with a frequency of 30% (Figures 4D,E). Other residues, including Tyr109, Asn145, Trp161, and Gly236, also contributed through hydrophobic and ionic interactions, further stabilizing the complex. The α2,6 SA itself formed several intramolecular hydrogen bonds, which contributed to its conformational stability (Figure 4E).

These findings suggest that the HA_198V_ mutation significantly enhances the binding affinity between the HA protein and α2,6 SA. The combination of hydrogen bonds, hydrophobic interactions, and water bridges forms a robust network that stabilizes the HA -α2,6 SA complex.

### MD simulation reveals enhanced binding affinity between HA198T and α2,6 SA

**Figure 5:**
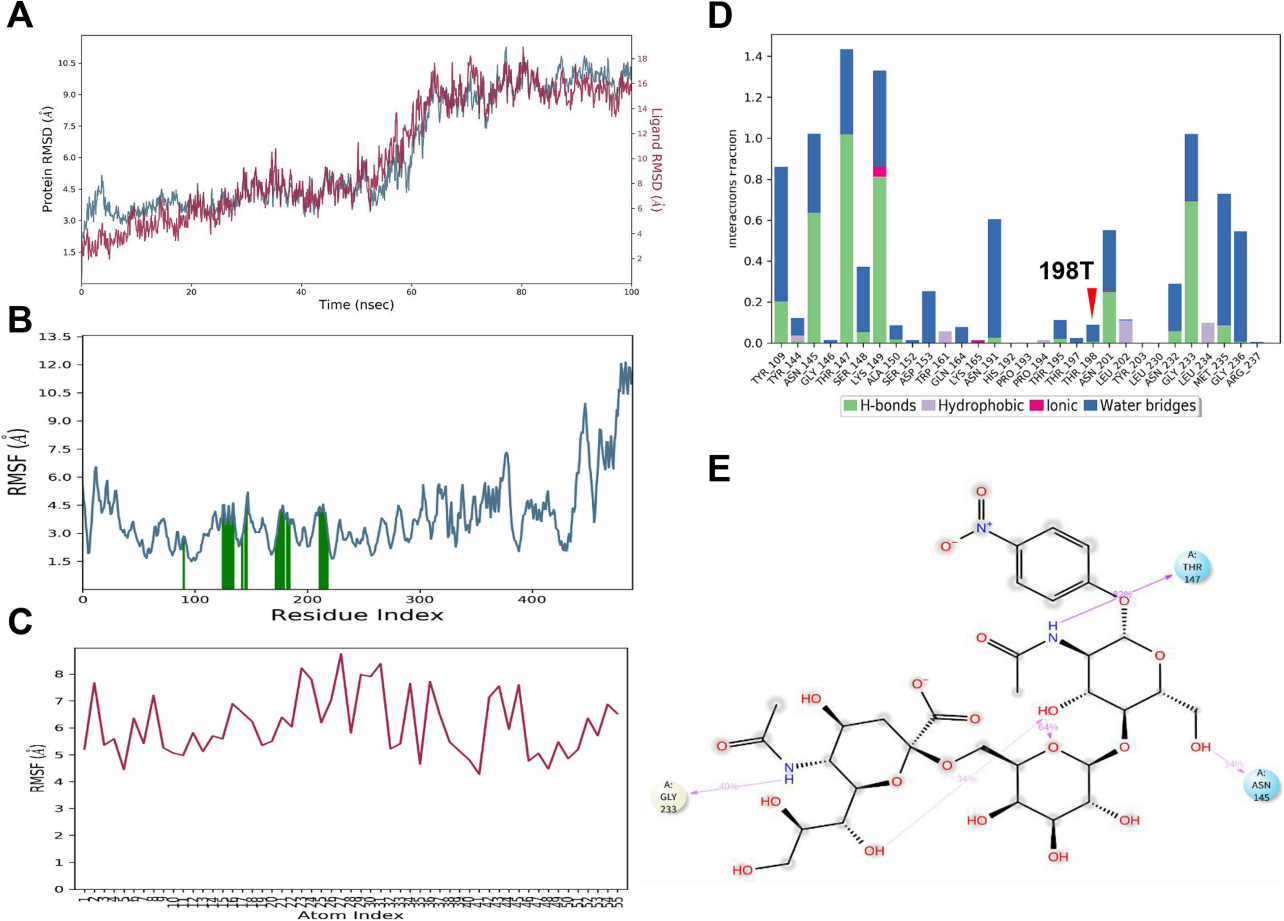
MD simulation results for 6’SLN binding to HA_198T_. **(A)** RMSD of HA_198T_ atoms (blue) and 6’SLN atoms (red) over 100 ns simulation time. **(B)** RMSF of HA_198T_ residues. Green bars indicate residues involved in 6’SLN binding. **(C)** RMSF of 6’SLN atoms over the simulation trajectory. **(D)** Interaction fractions of key residues with the 6’SLN, categorized by interaction type. The 198 T mutation site is indicated with a red arrow. **(E)** 2D representation of key interactions between 6’SLN and HA_198T_ residues at the end of the simulation. Hydrogen bonds are shown as purple dashed lines.

In the MD simulation of the HA_198T_ protein complexed with the α2,6 SA, the RMSD of the HA_198T_ protein stabilized at approximately 9.0 Å, while the α2,6 SA’s RMSD stabilized at around 16 Å after initial fluctuations (Figure 5A). These RMSD values indicate that both the protein and α2,6 SA underwent structural adjustments before reaching a stable conformation.

Further analysis using RMSF revealed that the HA_198T_ protein exhibited relatively low fluctuations, indicating minimal conformational changes during the binding process (Figure 5B). In contrast, the α2,6 SA displayed higher RMSF values, highlighting its dynamic and flexible nature (Figure 5C). This flexibility likely facilitated binding interactions with the protein, contributing to the formation of a more stable complex.

A total of 30 interactions were identified between the HA_198T_ protein and the α2,6 SA, with Thr147 playing a critical role, showing a hydrogen bond formation frequency of 92% (Figures 5D,E). Other residues, such as Asn145 and Gly233, also contributed significantly to the stability of the complex, reinforcing the hydrogen bonding network. Additionally, the α2,6 SA formed several intramolecular hydrogen bonds, further stabilizing its conformation and strengthening the overall α2,6 SA-protein complex (Figure 5E).

Overall, the HA_198T_ mutation enhances the binding affinity between the HA protein and the α2,6 SA. The hydrogen bond network, particularly involving Thr147, is crucial in stabilizing the complex. The flexibility of the α2,6 SA, along with its intramolecular interactions, plays a significant role in the stability and dynamic behavior of the α2,6 SA-protein interaction. These findings provide valuable insights into the molecular mechanisms underlying the increased receptor binding affinity due to the HA_198T_ mutation, with potential implications for future vaccine or antiviral drug development.

### MD simulation reveals unstable binding and dissociation between HA198A and α2,6 SA

**Figure 6:**
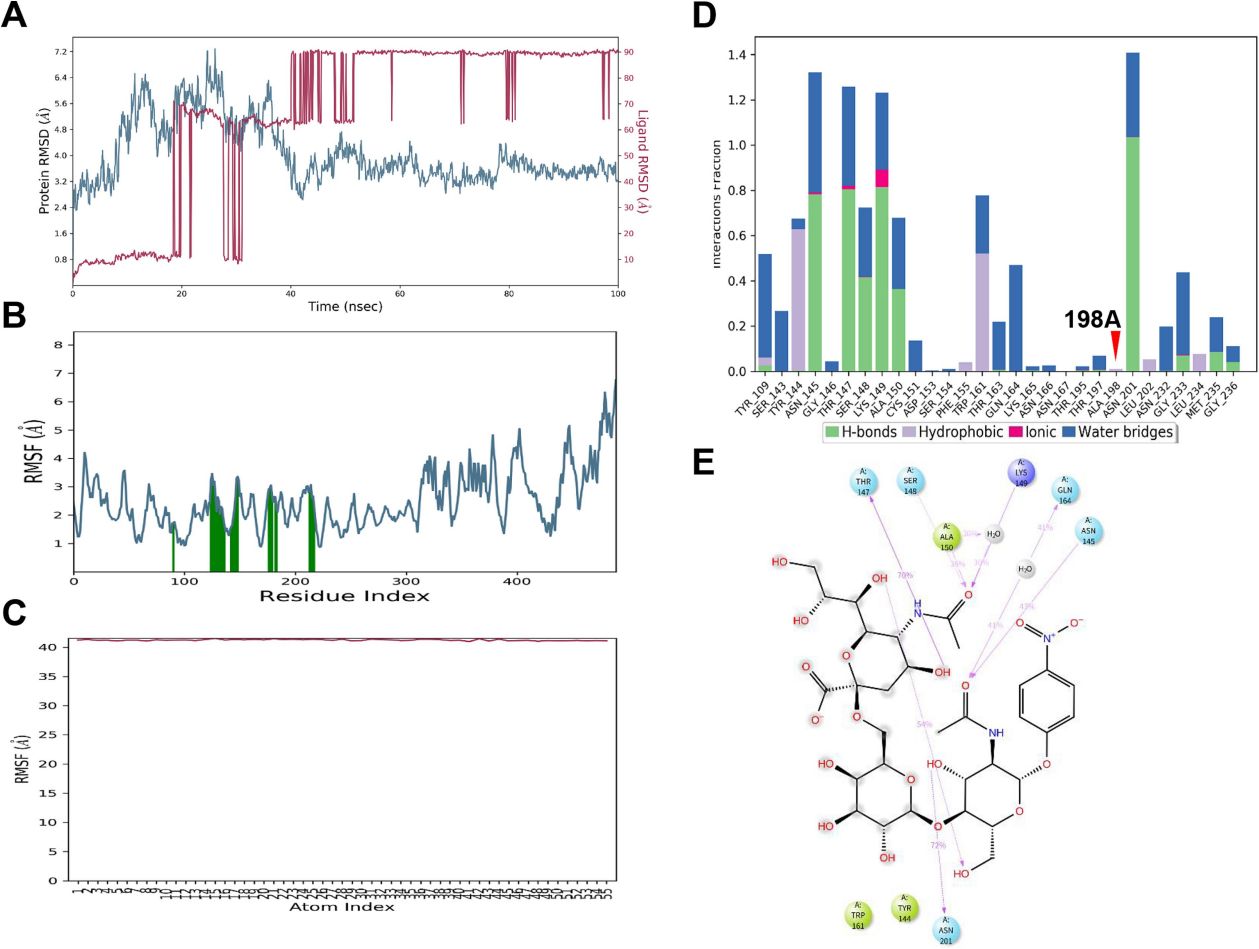
MD simulation results for 6’SLN and HA_198A_. **(A)** Time evolution of HA_198A_ RMSD (blue) and 6’SLN RMSD (red) during the 100 ns simulation. **(B)** RMSF of HA_198A_ residues. Green bars indicate regions of high flexibility. **(C)** RMSF of 6’SLN atoms, showing stable binding throughout the simulation. **(D)** Fraction of different types of interactions (hydrogen bonds, hydrophobic interactions, ionic interactions, and water bridges) between the HA_198A_ and 6’SLN for key residues. The red arrow indicates residue 198A. **(E)** 2D interaction diagram of the ligand with surrounding amino acid residues, showing hydrogen bonds, hydrophobic interactions, and water-mediated interactions.

During the molecular dynamics simulation, the HA_198A_ protein exhibited significant fluctuations. The RMSD of HA_198A_ eventually stabilized at approximately 4.0 Å, whereas the RMSD of the α2,6 SA fluctuated substantially, finally stabilizing near 90 Å (Figure 6A).

While RMSF of the HA_198A_ protein remained relatively small, the RMSF of the α2,6 SA was notably larger (Figures 6B,C), indicating greater movement of the α2,6 SA during the simulation. Despite the formation of 29 interactions, the α2,6 SA eventually moved away from the active binding pocket (Figures 6D,E).

These results suggest that the initial binding conformation between the α2,6 SA and the HA_198A_ protein was unstable, characterized by weak binding affinity. During the simulation, the α2,6 SA ultimately dissociated from the binding pocket, failing to maintain a stable interaction with the HA protein.

### MD simulation reveals moderate binding affinity between HA198V and α2,3 SA after initial instability

**Figure 7:**
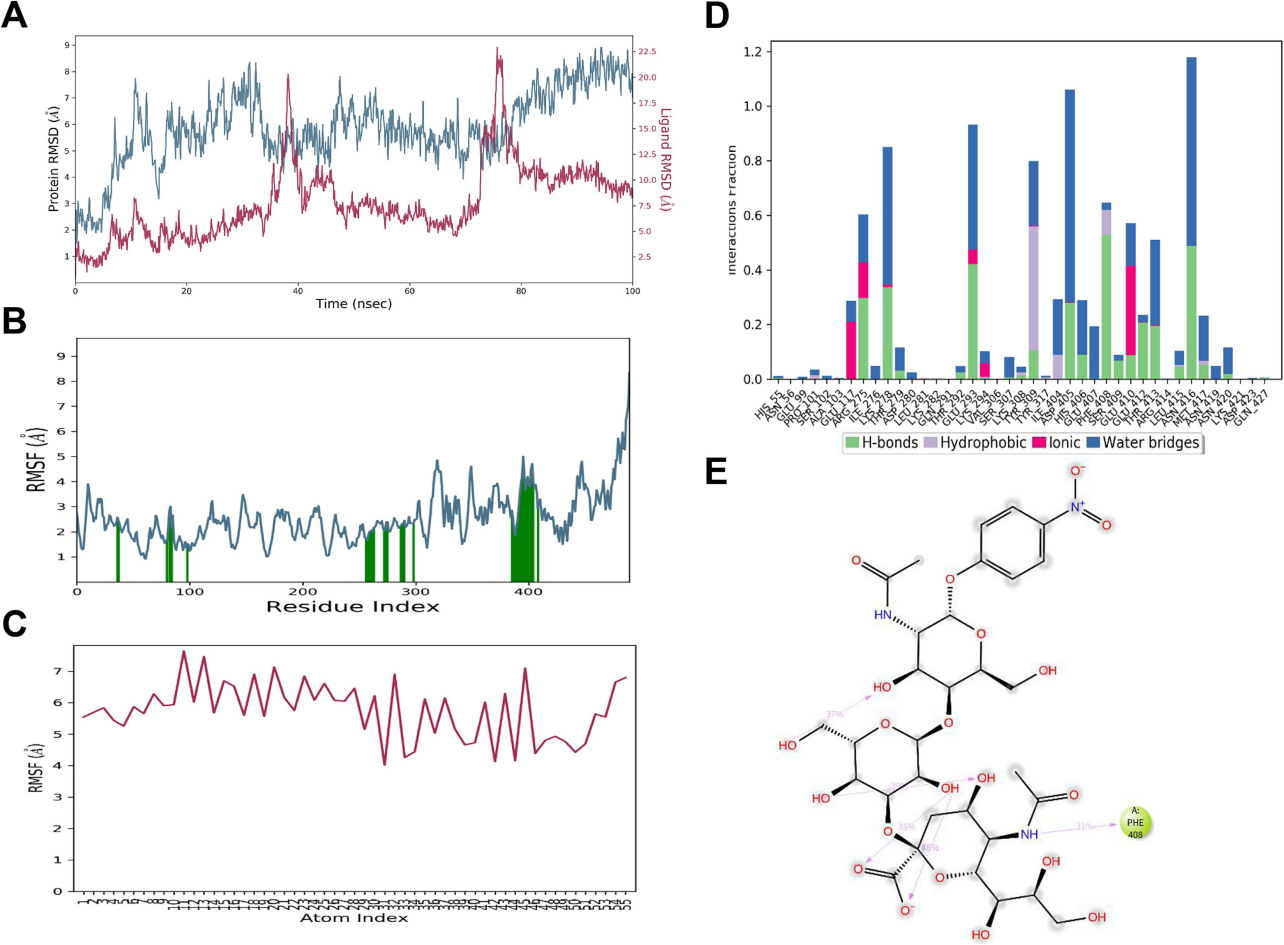
MD simulation results for 3’SLN and HA_198V_. **(A)** RMSD plot of HA_198V_ (blue) and 3’SLN (red) over 100 ns simulation time. **(B)** RMSF plot of HA_198V_ residues. Green bars indicate key binding site residues. **(C)** RMSF plot of 3’SLN atoms. **(D)** Interaction fraction analysis of HA_198V_-3’SLN contacts, categorized by interaction type. **(E)** 2D representation of the 3’SLN structure with key interacting residue (Phe408) highlighted.

During the MD simulation, the RMSD of the HA_198V_ protein stabilized at approximately 8.0 Å, while the RMSD of the α2,3 SA, after some fluctuations, settled around 10.0 Å (Figure 7A). The RMSF values for the amino acid residues interacting with the α2,3 SA, highlighted by green lines in Figure 7B, were relatively low, indicating minimal conformational changes in these regions during the binding process. In contrast, the RMSF values for the atoms in the α2,3 SA were relatively high, reflecting substantial fluctuations in the α2,3 SA ‘s structure (Figure 7C). These findings suggest that the initial binding conformation was unstable, though the system eventually achieved a more stable conformation after significant fluctuations.

The HA_198V_ protein and the α2,3 SA formed 41 interactions, with Phe408 playing a pivotal role by forming hydrogen bonds with a 31% frequency, highlighting its importance in the binding process. Other interaction sites contributed via hydrogen bonds and water bridges, although these interactions were comparatively weak (Figures 7D,E). Additionally, the α2,3 SA formed several intramolecular hydrogen bonds that helped stabilize its conformation during binding (Figure 7E).

Overall, these results indicate that the HA_198V_ protein and α2,3 SA optimized their binding conformation through the simulation, achieving a more stable structure with moderate binding affinity.

### MD simulation confirms weak binding characteristics between HA198T and α2,3 SA

**Figure 8:**
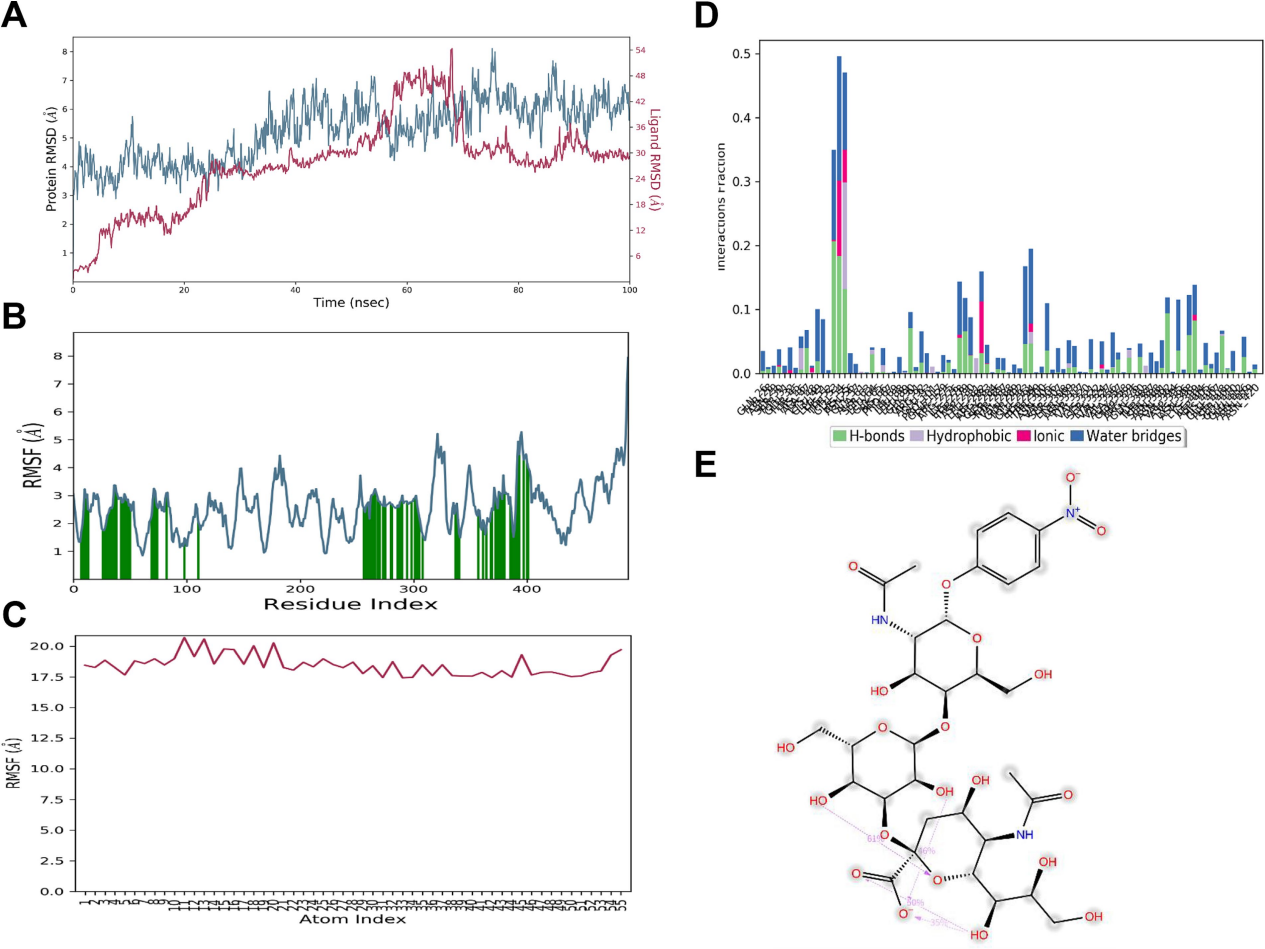
MD simulation results for 3’SLN and HA_198T_ interaction. **(A)** Time evolution of HA_198T_ RMSD (blue) and 3’SLN RMSD (red) during the 100 ns simulation. **(B)** Per-residue RMSF of the HA_198T_. Green bars indicate residues involved in 3’SLN binding. **(C)** Per-atom RMSF of the 3’SLN. **(D)** Fraction of simulation time different types of interactions (hydrogen bonds, hydrophobic, ionic, and water bridges) were maintained between the HA_198T_ and 3’SLN. **(E)** 2D representation of the key interactions between 3’SLN and HA_198T_, showing hydrogen bonds (dotted lines) and other non-covalent interactions.

During the MD simulation, the HA_198T_ protein exhibited minimal fluctuations, with its RMSD stabilizing at approximately 6 Å. In contrast, the RMSD of the α2,3 SA displayed significant variability before eventually stabilizing at around 30 Å (Figure 8A). The RMSF values for the HA_198T_ protein were relatively low, indicating limited conformational changes, while the α2,3 SA exhibited much higher RMSF values, suggesting considerable fluctuations in the α2,3 SA’s structure throughout the simulation (Figures 8B,C). This pattern suggests that the initial conformation of the HA_198T_ protein and α2,3 SA complex was unstable, with the α2,3 SA gradually moving away from the binding pocket during the course of the simulation.

Although a total of 91 interactions were formed between the protein and α2,3 SA, none of these interactions had frequencies exceeding 30% (Figure 8D), and only those above this threshold are shown in Figures 8D,E. The absence of strong, consistent interactions indicates a weak binding affinity between the HA_198T_ protein and the α2,3 SA. Consequently, the α2,3 SA was unable to maintain stable binding within the protein’s receptor pocket, which is consistent with its detachment from the binding site.

### MD simulation reveals high-affinity stable binding between HA198A and α2,3 SA

**Figure 9:**
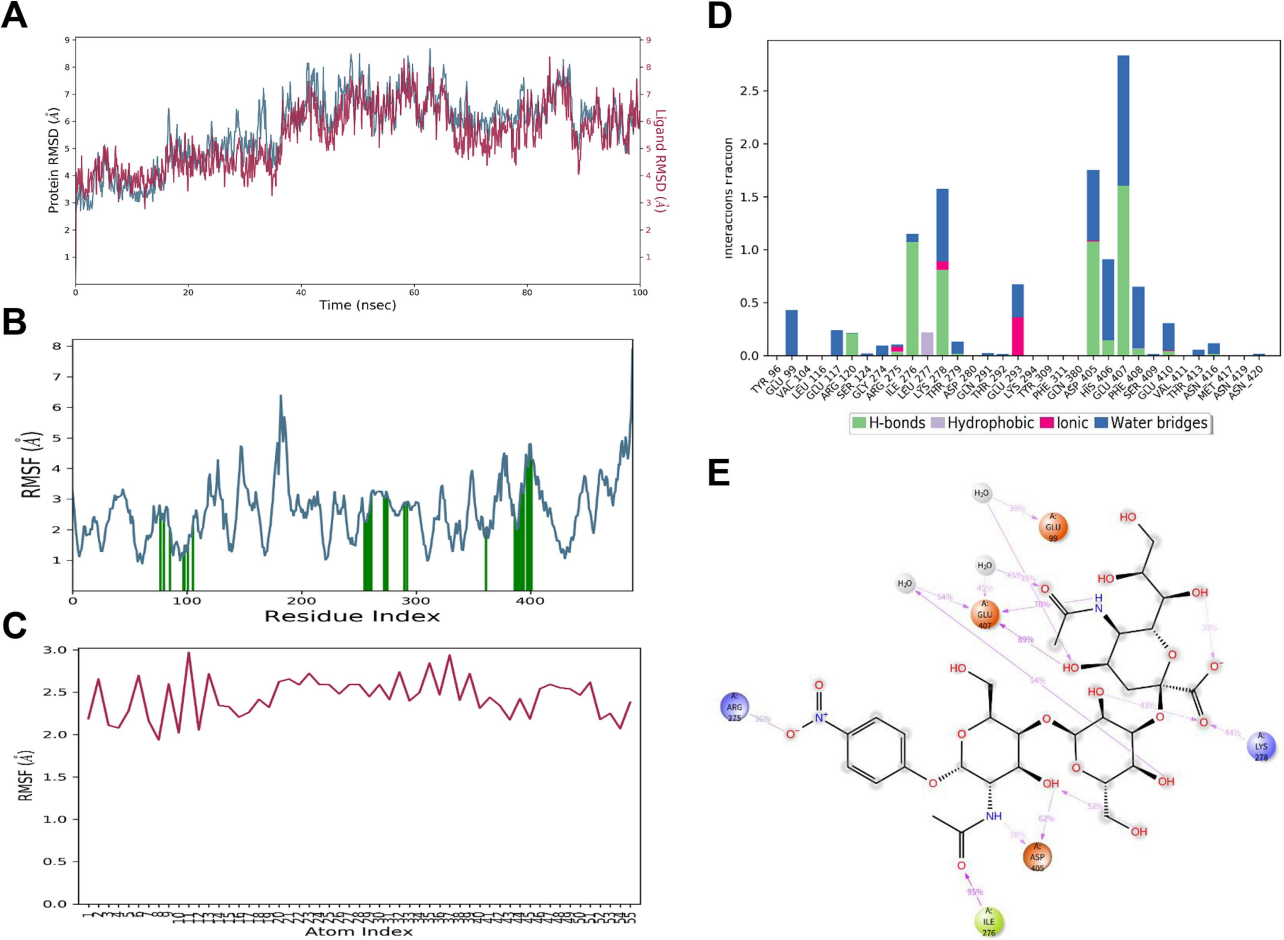
MD simulation results for 3’SLN and HA_198A_ interaction. **(A)** RMSD of HA_198A_ (blue) and 3’SLN (red) over the 100 ns simulation time. **(B)** RMSF of HA_198A_ residues. Green bars indicate key binding site residues. **(C)** RMSF of 3’SLN atoms over the simulation period. **(D)** Interaction fraction of different types of bonds (hydrogen bonds, hydrophobic interactions, ionic bonds, and water bridges) between the 3’SLN and specific HA_198A_ residues. **(E)** 2D interaction diagram showing key binding interactions between 3’SLN and HA_198A_ residues at the end of the simulation. Dotted lines represent different types of interactions: pink for hydrogen bonds, green for *π*-π stacking, and blue for water bridges.

During the MD simulation, both the HA_198A_ protein and the α2,3 SA demonstrated high stability. The RMSD of the HA_198A_ protein stabilized at approximately 6.0 Å, and after initial fluctuations, the RMSD of the α2,3 SA also stabilized at around 6.0 Å (Figure 9A). The RMSF values for the interacting amino acid residues of HA_198A_ (indicated in green in Figure 9B) and the α2,3 SA atoms showed minimal fluctuations overall (Figures 9B,C), indicating that both the protein and the α2,3 SA had relatively stable initial conformations. After some dynamic movements, a more stable binding conformation was formed.

The HA_198A_ protein and the α2,3 SA formed a total of 33 interactions. Notably, the hydrogen bond and water bridge interaction frequencies of Glu407 reached 159 and 99%, respectively, underscoring the critical role of Glu407 in the binding process (Figures 9D,E). Other important residues, including Glu99, Arg275, Ile276, Lys278, and Asp405, also contributed significantly to maintaining the complex’s stability (Figures 9D,E). Additionally, several intramolecular hydrogen bonds were formed within the α2,3 SA, further stabilizing its binding conformation (Figure 9E).

These results suggest that the α2,3 SA and HA_198A_ protein optimized their initial binding conformations during molecular dynamics simulations, resulting in a more stable complex with high binding affinity.

In summary, MD simulations provided insights into the molecular interactions and conformational changes governing the binding affinity between the HA protein and sialic acids, which are critical for viral receptor specificity. The binding affinity between the 198th residue of the HA protein and the α2,6 SA decreased in the order of HA_198V_, HA_198T_, and HA_198A_. Conversely, for the α2,3 SA, the binding affinity increased in the order of HA_198T_, HA_198V_, and HA_198A_. Furthermore, when interacting with the α2,6 SA, the 198th residue primarily engaged through the formation of water bridges. In contrast, the 198th residue did not directly participate in interactions with the α2,3 SA, suggesting a differential role of this residue in modulating receptor specificity based on SA type.

## Discussion

The ongoing evolution of the H9N2 AIV has led to a mismatch between epidemic and vaccine strains, contributing significantly to immune failure and periodic outbreaks. Recent years have seen extensive documentation of the diversity and high mutability of amino acids at position 198 on the HA protein, closely associated with the adaptive evolution of H9N2 AIV (11, 31–34). This study aimed to elucidate the molecular mechanisms by which high-frequency amino acids at position 198 on the HA protein influence receptor binding affinity and specificity, providing new insights into the adaptive evolution patterns, antigenic variation mechanisms, and cross-species transmission potential of H9N2 AIV.

Our analysis revealed that between 2013 and 2021, 79.36% of H9N2 AIV strains in China contained the 190 T/V mutation, corroborating and extending the findings of Sun et al. (35), who reported an increase from 22.9% before 1999 to 57.2% between 2013 and 2019. This significant increase could explain the rising number of H9N2 avian influenza viruses with enhanced mammalian infection potential, potentially contributing to the observed increase in human infections. The public health implications of this trend are considerable, highlighting the need for enhanced surveillance strategies and the development of more effective vaccines that can address the evolving nature of these viruses. The differential binding patterns we observed between HA variants and non-specific antibodies provide new insights into H9N2 immune escape mechanisms. Our findings that the 198 V mutation reduces non-specific antibody binding while enhancing receptor binding avidity suggests a mechanism where increased receptor competition facilitates antibody escape, consistent with previous observations in other influenza subtypes (15, 16). This is particularly significant given that position 198 contributes to both receptor binding and antigenic recognition (17). The enhanced receptor binding associated with 198 V mutation may provide these variants with a competitive advantage in evading host immunity while maintaining efficient cell entry.

Furthermore, we observed a negative correlation between the prevalence trends of the 198V and 198T mutations. This intriguing finding could potentially reflect evolutionary trade-offs in viral fitness. While both mutations enhance binding to human-type receptors, 198 V appears to confer notably higher receptor binding affinity. Based on previous studies (15), such increased binding avidity might drive antigenic drift, which could explain our observation of reduced polyclonal antibody binding to 198 V variants. Additionally, this stronger binding might potentially disrupt the optimal HA-NA balance required for efficient transmission. In contrast, 198 T’s moderate binding affinity could theoretically maintain better functional balance while providing sufficient receptor engagement. We speculate that this potential trade-off between immune escape and transmission efficiency might contribute to their opposing evolutionary patterns.

As previously reported (14), the HA_198V_ mutation in the H9N2 virus promoted viral escape from polyclonal antibody binding by enhancing receptor-binding activity, consistent with the findings of this study. Additionally, our results demonstrate that mutations at HA position 198 significantly influence receptor binding preferences and affinities. Specifically, HA_198V_ and HA_198T_ variants exhibit higher affinity for 6’SLN, a receptor more prevalent in the upper respiratory tract of mammals (36), compared to HA_198A_. Conversely, the HA_198A_ variant shows a stronger preference for 3’SLN. These differential binding patterns, consistently demonstrated in both RDE and solid-phase binding assays, align with previous reports of HA_198V/T_ mutations promoting mammalian adaptation (31). Moreover, the observed differences in non-specific antibody binding among HA_198V_, HA_198T_, and HA_198A_ variants suggest complex interactions between receptor binding and antibody recognition. These findings have significant implications for vaccine design and efficacy, particularly in light of the ongoing challenge of antigenic drift in H9N2 AIV. The altered antibody binding profiles associated with different HA_198_ variants underscore the need for vaccine strategies that can induce broadly specific antibodies or that can be quickly updated to match circulating strains.

To further elucidate the mechanisms underlying changes in receptor-binding strength and specificity caused by HA_198_ amino acid mutations, we employed molecular docking and molecular dynamics simulations. These computational approaches revealed that mutations such as HA_198V_, HA_198T_, and HA_198A_ induce conformational changes in the receptor-binding pocket, significantly altering the interactions between the H9N2 virus and both α2,6 SA and α2,3 SA receptors. The molecular docking analysis revealed small but consistent differences in binding energies between variants (−5.5 to −6.1 kcal/mol), which, while subtle, align well with our solid-phase binding assay results showing differential receptor binding patterns. The predicted binding modes and energetic differences are further validated by our 100-ns MD simulations, which demonstrate stable protein-ligand interactions and consistent conformational differences, providing a comprehensive molecular basis for the observed receptor binding preferences.

Specifically, during MD simulations, the HA_198V_ and HA_198T_ mutations enhanced binding stability with 6’SLN, while HA_198A_ formed a more stable complex with 3’SLN. We also identified key residues, such as Thr147, Glu199, and Tyr109, that play crucial roles in receptor binding by forming stable hydrogen bonds and water bridges with sialic acids. Notably, residue 198 primarily interacts with α2,6 SA through water bridges, while showing minimal direct involvement in binding with α2,3 SA. These molecular-level changes could translate to broader phenotypic effects, such as altered transmissibility or virulence in different host species. For instance, the enhanced stability of HA_198V_ and HA_198T_ with 6’SLN might contribute to more efficient viral entry and replication in mammalian cells, potentially increasing the risk of zoonotic transmission. Conversely, the preference of HA_198A_ for 3’SLN might optimize the virus for replication in avian hosts. Understanding these structure–function relationships at the molecular level provides valuable insights into the mechanisms of host adaptation and could inform the development of targeted antiviral strategies.

This study underscores the advantages of molecular docking and MD simulations in analyzing virus-receptor interactions. Compared to traditional biological assays, these computational techniques offer more direct visualization of the dynamic interactions between viral proteins and receptors, revealing molecular changes and their biological significance. These tools provide a powerful means to study the adaptive evolution and antigenic drift of the H9N2 virus, allowing for rapid assessment of the potential impact of newly identified HA mutations on receptor binding. However, it is important to acknowledge the limitations of these computational approaches. While they provide valuable insights into molecular interactions, they may not fully capture the complexity of the cellular environment or the potential influence of other viral and host factors. Therefore, these computational studies should be viewed as complementary to experimental approaches. Integrating computational predictions with *in vitro* and *in vivo* experimental validation can provide a more comprehensive understanding of virus-host interactions and enhance the reliability of our findings.

In conclusion, this study combined experimental data with computational simulations to elucidate how mutations at residue 198 of the HA protein regulate receptor-binding affinity, specificity, and antibody recognition, driving viral antigenic drift and host adaptation. These findings offer valuable insights into the underlying molecular mechanisms of H9N2 AIV evolution and adaptation.

## Data Availability

The original contributions presented in the study are included in the article/supplementary material, further inquiries can be directed to the corresponding authors.

## Footnotes

^1^ https://www.deshawresearch.com

## References

[1] Sun Y Liu J. H9N2 influenza virus in China: a cause of concern. Protein Cell. (2015) 6:18–25. doi: 10.1007/s13238-014-0111-7, PMID: 25384439 PMC4286136

[2] Kishida N Sakoda Y Eto M Sunaga Y Kida H. Co-infection of staphylococcus aureus or haemophilus paragallinarum exacerbates H9N2 influenza a virus infection in chickens. Arch Virol. (2004) 149:2095–104. doi: 10.1007/s00705-004-0372-1, PMID: 15503199

[3] Wang J Li Y Yin Y. Respiratory phagocytes are implicated in enhanced colibacillosis in chickens co-infected with influenza virus H9N2 and escherichia coli. Br Poult Sci. (2018) 59:160–5. doi: 10.1080/00071668.2017.1406061, PMID: 29148834

[4] Wang X Wang H Zhang S Shang H Wang C Zhou F. The role of transforming growth factor beta-1 protein in escherichia coli secondary infection induced by H9N2 avian influenza virus in chickens. Microb Pathog. (2023) 175:105983. doi: 10.1016/j.micpath.2023.105983 36641002

[5] Pu J Wang S Yin Y Zhang G Carter RA Wang J. Evolution of the H9N2 influenza genotype that facilitated the genesis of the novel H7N9 virus. Proc Natl Acad Sci USA. (2015) 112:548–53. doi: 10.1073/pnas.1422456112, PMID: 25548189 PMC4299237

[6] Ding S Zhou J Xiong J du X Yang W Huang J. Continued evolution of H10N3 influenza virus with adaptive mutations poses an increased threat to mammals. Virol Sin. (2024) 39:546–55. doi: 10.1016/j.virs.2024.06.005, PMID: 38871182 PMC11401466

[7] Li B Su G Xiao C Zhang J Li H Sun N. The PB2 co-adaptation of H10N8 avian influenza virus increases the pathogenicity to chickens and mice. Transbound Emerg Dis. (2022) 69:1794–803. doi: 10.1111/tbed.14157, PMID: 34008327

[8] Mao Q Zhou S Liu S Peng C Yin X Li J. Emergence of novel reassortant H3N3 avian influenza viruses with increased pathogenicity in chickens in 2023. Emerg Microb Infect. (2024) 13:2287683. doi: 10.1080/22221751.2023.2287683, PMID: 37990831 PMC10795584

[9] Li P Niu M Li Y Xu M Zhao T Cao X. Human infection with H3N8 avian influenza virus: a novel H9N2-original reassortment virus. J Infect. (2022) 85:e187–9. doi: 10.1016/j.jinf.2022.08.033, PMID: 36058414

[10] Koel BF Burke DF Bestebroer TM van der Vliet S Zondag GCM Vervaet G. Substitutions near the receptor binding site determine major antigenic change during influenza virus evolution. Science. (2013) 342:976–9. doi: 10.1126/science.1244730 24264991

[11] Zhu R Xu D Yang X Zhang J Wang S Shi H. Genetic and biological characterization of H9N2 avian influenza viruses isolated in China from 2011 to 2014. PLoS One. (2018) 13:e0199260. doi: 10.1371/journal.pone.0199260, PMID: 29969454 PMC6029760

[12] Su H Zhao Y Zheng L Wang S Shi H Liu X. Effect of the selection pressure of vaccine antibodies on evolution of H9N2 avian influenza virus in chickens. AMB Express. (2020) 10:98. doi: 10.1186/s13568-020-01036-0, PMID: 32462233 PMC7253569

[13] Jin H Wang W Yang X Su H Fan J Zhu R. Evolution of H9N2 avian influenza virus in embryonated chicken eggs with or without homologous vaccine antibodies. BMC Vet Res. (2018) 14:71. doi: 10.1186/s12917-018-1391-6, PMID: 29510698 PMC5840701

[14] Zhu R Xu S Sun W Li Q Wang S Shi H. HA gene amino acid mutations contribute to antigenic variation and immune escape of H9N2 influenza virus. Vet Res. (2022) 53:43. doi: 10.1186/s13567-022-01058-5, PMID: 35706014 PMC9202205

[15] Hensley SE Das SR Bailey AL Schmidt LM Hickman HD Jayaraman A. Hemagglutinin receptor binding avidity drives influenza a virus antigenic drift. Science. (2009) 326:734–6. doi: 10.1126/science.1178258, PMID: 19900932 PMC2784927

[16] Sealy JE Yaqub T Peacock TP Chang P Ermetal B Clements A. Association of increased receptor-binding avidity of influenza a(H9N2) viruses with escape from antibody-based immunity and enhanced zoonotic potential. Emerg Infect Dis. (2018) 25:63–72. doi: 10.3201/eid2501.180616, PMID: 30561311 PMC6302589

[17] Peacock TP Sealy JE Harvey WT Benton DJ Reeve R Iqbal M. Genetic determinants of receptor-binding preference and zoonotic potential of H9N2 avian influenza viruses. J Virol. (2021) 95:e01651–20. doi: 10.1128/JVI.01651-20, PMID: 33268517 PMC8092835

[18] Koehler M Delguste M Sieben C Gillet L Alsteens D. Initial step of virus entry: virion binding to cell-surface glycans. Annu Rev Virol. (2020) 7:143–65. doi: 10.1146/annurev-virology-122019-070025, PMID: 32396772

[19] Overeem NJ van der Vries E Huskens J. A dynamic, supramolecular view on the multivalent interaction between influenza virus and host cell. Small. (2021) 17:e2007214. doi: 10.1002/smll.202007214, PMID: 33682339

[20] Zhao C Pu J. Influence of host sialic acid receptors structure on the host specificity of influenza viruses. Viruses. (2022) 14:2141. doi: 10.3390/v14102141, PMID: 36298694 PMC9608321

[21] Vora J Athar M Sinha S Jha PC Shrivastava N. Binding insight of anti-HIV phytocompounds with prime targets of HIV: a molecular dynamics simulation analysis. Curr HIV Res. (2020) 18:132–41. doi: 10.2174/1570162X18666200129112509, PMID: 31995010

[22] Durrant JD McCammon JA. Molecular dynamics simulations and drug discovery. BMC Biol. (2011) 9:71. doi: 10.1186/1741-7007-9-71, PMID: 22035460 PMC3203851

[23] Hao X Hu J Wang J Xu J Cheng H Xu Y. Reassortant H5N1 avian influenza viruses containing PA or NP gene from an H9N2 virus significantly increase the pathogenicity in mice. Vet Microbiol. (2016) 192:95–101. doi: 10.1016/j.vetmic.2016.07.002, PMID: 27527770

[24] Peng Q Zhu R Wang X Shi H Bellefleur M Wang S. Impact of the variations in potential glycosylation sites of the hemagglutinin of H9N2 influenza virus. Virus Genes. (2019) 55:182–90. doi: 10.1007/s11262-018-1623-7, PMID: 30594968 PMC6458969

[25] Chambers BS Parkhouse K Ross TM Alby K Hensley SE. Identification of hemagglutinin residues responsible for H3N2 antigenic drift during the 2014–2015 influenza season. Cell Rep. (2015) 12:1–6. doi: 10.1016/j.celrep.2015.06.005, PMID: 26119736 PMC4487778

[26] Zhang N Quan K Chen Z Hu Q Nie M Xu N. The emergence of new antigen branches of H9N2 avian influenza virus in China due to antigenic drift on hemagglutinin through antibody escape at immunodominant sites. Emerg Microbes Infect. (2023) 12:2246582. doi: 10.1080/22221751.2023.2246582, PMID: 37550992 PMC10444018

[27] Gao R Gu M Liu K Li Q Li J Shi L. T160A mutation-induced deglycosylation at site 158 in hemagglutinin is a critical determinant of the dual receptor binding properties of clade 2.3.4.4 H5NX subtype avian influenza viruses. Vet Microbiol. (2018) 217:158–66. doi: 10.1016/j.vetmic.2018.03.018, PMID: 29615249

[28] O’Boyle NM Banck M James CA Morley C Vandermeersch T Hutchison GR. Open babel: an open chemical toolbox. J Cheminform. (2011) 3:33. doi: 10.1186/1758-2946-3-33, PMID: 21982300 PMC3198950

[29] Eberhardt J Santos-Martins D Tillack AF Forli S. AutoDock vina 1.2.0: new docking methods, expanded force field, and python bindings. J Chem Inf Model. (2021) 61:3891–8. doi: 10.1021/acs.jcim.1c00203, PMID: 34278794 PMC10683950

[30] Trott O Olson AJ. AutoDock vina: improving the speed and accuracy of docking with a new scoring function, efficient optimization and multithreading. J Comput Chem. (2010) 31:455–61. doi: 10.1002/jcc.21334, PMID: 19499576 PMC3041641

[31] Amin F Mukhtar N Ali M Shehzad R Ayub S Aslam A. Mapping genetic markers associated with antigenicity and host range in H9N2 influenza a viruses infecting poultry in Pakistan. Avian Dis. (2024) 68:43–51. doi: 10.1637/aviandiseases-D-23-00029, PMID: 38687107

[32] Gao X Wang N Chen Y Gu X Huang Y Liu Y. Sequence characteristics and phylogenetic analysis of H9N2 subtype avian influenza a viruses detected from poultry and the environment in China, 2018. PeerJ. (2021) 9:e12512. doi: 10.7717/peerj.12512, PMID: 35036116 PMC8697764

[33] Yang F Xiao Y Liu F Yao H Wu N Wu H. Molecular characterization and antigenic analysis of reassortant H9N2 subtype avian influenza viruses in eastern China in 2016. Virus Res. (2021) 306:198577. doi: 10.1016/j.virusres.2021.198577, PMID: 34560182

[34] Xia J Li Y-X Dong M-Y Guo ZW Luo YW Li NL. Evolution of prevalent H9N2 subtype of avian influenza virus during 2019 to 2022 for the development of a control strategy in China. Poult Sci. (2023) 102:102957. doi: 10.1016/j.psj.2023.102957, PMID: 37573848 PMC10448327

[35] Sun X Belser JA Maines TR. Adaptation of H9N2 influenza viruses to mammalian hosts: a review of molecular markers. Viruses. (2020) 12:541. doi: 10.3390/v12050541, PMID: 32423002 PMC7290818

[36] Sriwilaijaroen N Kondo S Yagi H Takemae N Saito T Hiramatsu H. N-glycans from porcine trachea and lung: predominant NeuAcα2-6Gal could be a selective pressure for influenza variants in favor of human-type receptor. PLoS One. (2011) 6:e16302. doi: 10.1371/journal.pone.0016302, PMID: 21347401 PMC3036579

